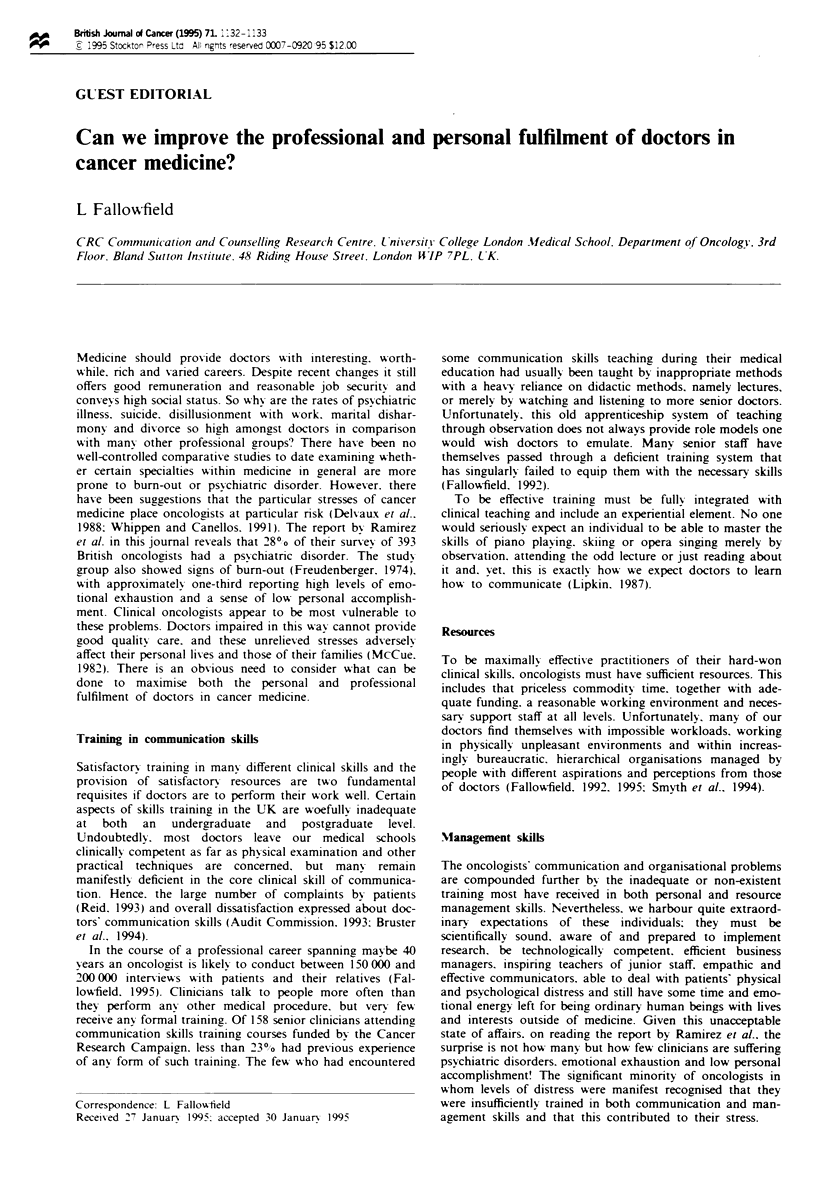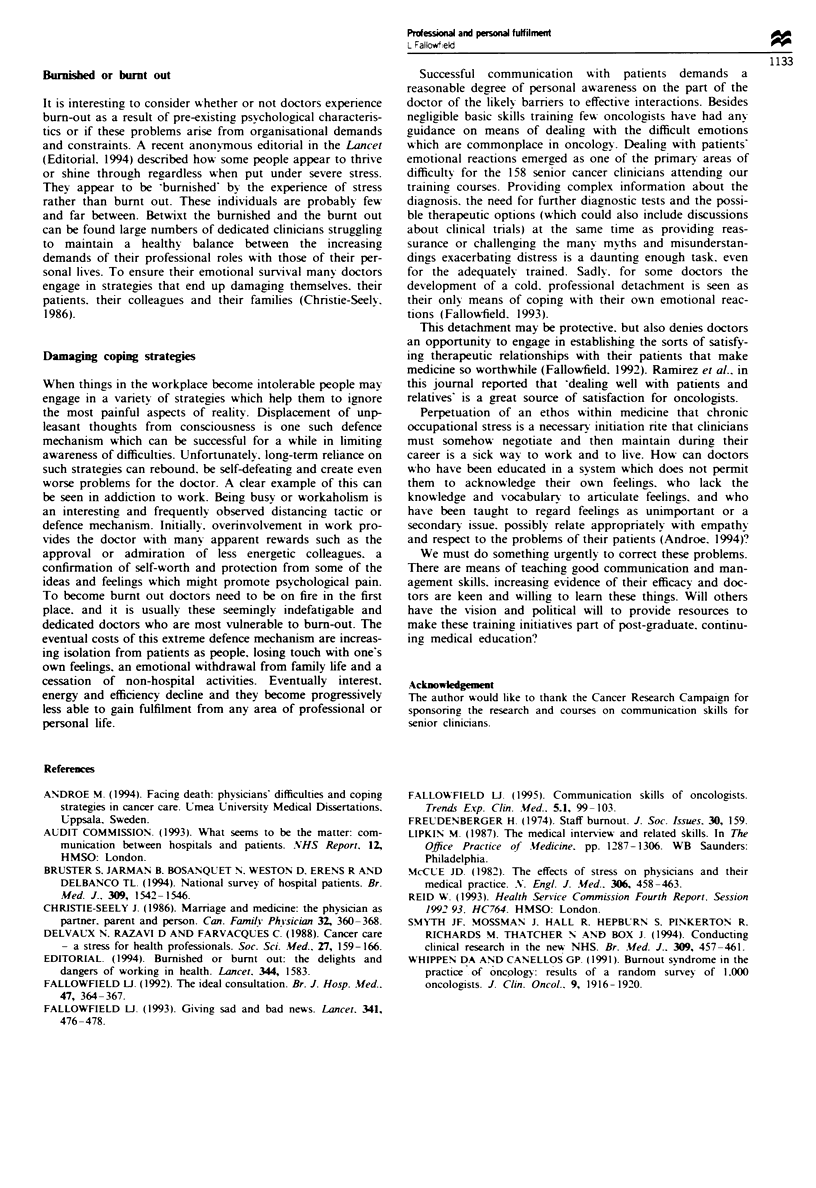# Can we improve the professional and personal fulfillment of doctors in cancer medicine?

**DOI:** 10.1038/bjc.1995.220

**Published:** 1995-06

**Authors:** L. Fallowfield


British Journal of Cancer (1995) 71, 1132–1133

© 1995 Stockton Press Ltd All rights reserved 0007-0920/95 $12.00

GUEST EDITORIAL

### Can we improve the professional and personal fulfilment of doctors in cancer medicine?

L Fallowfield

*CRC Communication and Counselling Research Centre, University College London Medical School, Department of Oncology, 3rd Floor, Bland Sutton Institute, 48 Riding House Street, London W1P 7PL, UK.*

Medicine should provide doctors with interesting, worthwhile, rich and varied careers. Despite recent changes it still offers good remuneration and reasonable job security and conveys high social status. So why are the rates of psychiatric illness, suicide, disillusionment with work, marital disharmony and divorce so high amongst doctors in comparison with many other professional groups? There have been no well-controlled comparative studies to date examining whether certain specialties within medicine in general are more prone to burn-out or psychiatric disorder. However, there have been suggestions that the particular stresses of cancer medicine place oncologists at particular risk (Delvaux *et al.*, 1988; Whippen and Canellos, 1991). The report by Ramirez *et al.* in this journal reveals that 28% of their survey of 393 British oncologists had a psychiatric disorder. The study group also showed signs of burn-out (Freudenberger, 1974), with approximately one-third reporting high levels of emotional exhaustion and a sense of low personal accomplishment. Clinical oncologists appear to be most vulnerable to these problems. Doctors impaired in this way cannot provide good quality care, and these unrelieved stresses adversely affect their personal lives and those of their families (McCue, 1982). There is an obvious need to consider what can be done to maximise both the personal and professional fulfilment of doctors in cancer medicine.

### Training in communication skills

Satisfactory training in many different clinical skills and the provision of satisfactory resources are two fundamental requisites if doctors are to perform their work well. Certain aspects of skills training in the UK are woefully inadequate at both an undergraduate and postgraduate level. Undoubtedly, most doctors leave our medical schools clinically competent as far as physical examination and other practical techniques are concerned, but many remain manifestly deficient in the core clinical skill of communication. Hence, the large number of complaints by patients (Reid, 1993) and overall dissatisfaction expressed about doctors' communication skills (Audit Commission, 1993; Bruster *et al.*, 1994).

In the course of a professional career spanning maybe 40 years an oncologist is likely to conduct between 150 000 and 200 000 interviews with patients and their relatives (Fallowfield, 1995). Clinicians talk to people more often than they perform any other medical procedure, but very few receive any formal training. Of 158 senior clinicians attending communication skills training courses funded by the Cancer Research Campaign, less than 23% had previous experience of any form of such training. The few who had encountered some communication skills teaching during their medical education had usually been taught by inappropriate methods with a heavy reliance on didactic methods, namely lectures, or merely by watching and listening to more senior doctors. Unfortunately, this old apprenticeship system of teaching through observation does not always provide role models one would wish doctors to emulate. Many senior staff have themselves passed through a deficient training system that has singularly failed to equip them with the necessary skills (Fallowfield, 1992).

To be effective training must be fully integrated with clinical teaching and include an experiential element. No one would seriously expect an individual to be able to master the skills of piano playing, skiing or opera singing merely by observation, attending the odd lecture or just reading about it and, yet, this is exactly how we expect doctors to learn how to communicate (Lipkin, 1987).

### Resources

To be maximally effective practitioners of their hard-won clinical skills, oncologists must have sufficient resources. This includes that priceless commodity time, together with adequate funding, a reasonable working environment and necessary support staff at all levels. Unfortunately, many of our doctors find themselves with impossible workloads, working in physically unpleasant environments and within increasingly bureaucratic, hierarchical organisations managed by people with different aspirations and perceptions from those of doctors (Fallowfield, 1992, 1995; Smyth *et al.*, 1994).

### Management skills

The oncologists' communication and organisational problems are compounded further by the inadequate or non-existent training most have received in both personal and resource management skills. Nevertheless, we harbour quite extraordinary expectations of these individuals; they must be scientifically sound, aware of and prepared to implement research, be technologically competent, efficient business managers, inspiring teachers of junior staff, empathic and effective communicators, able to deal with patients' physical and psychological distress and still have some time and emotional energy left for being ordinary human beings with lives and interests outside of medicine. Given this unacceptable state of affairs, on reading the report by Ramirez *et al.*, the surprise is not how many but how few clinicians are suffering psychiatric disorders, emotional exhaustion and low personal accomplishment! The significant minority of oncologists in whom levels of distress were manifest recognised that they were insufficiently trained in both communication and management skills and that this contributed to their stress.

Correspondence: L Fallowfield
Received 27 January 1995; accepted 30 January 1995

### Burnished or burnt out

It is interesting to consider whether or not doctors experience burn-out as a result of pre-existing psychological characteristics or if these problems arise from organisational demands and constraints. A recent anonymous editorial in the *Lancet* (Editorial, 1994) described how some people appear to thrive or shine through regardless when put under severe stress. They appear to be 'burnished' by the experience of stress rather than burnt out. These individuals are probably few and far between. Betwixt the burnished and the burnt out can be found large numbers of dedicated clinicians struggling to maintain a healthy balance between the increasing demands of their professional roles with those of their personal lives. To ensure their emotional survival many doctors engage in strategies that end up damaging themselves, their patients, their colleagues and their families (Christie-Seely, 1986).

### Damaging coping strategies

When things in the workplace become intolerable people may engage in a variety of strategies which help them to ignore the most painful aspects of reality. Displacement of unpleasant thoughts from consciousness is one such defence mechanism which can be successful for a while in limiting awareness of difficulties. Unfortunately, long-term reliance on such strategies can rebound, be self-defeating and create even worse problems for the doctor. A clear example of this can be seen in addiction to work. Being busy or workaholism is an interesting and frequently observed distancing tactic or defence mechanism. Initially, overinvolvement in work provides the doctor with many apparent rewards such as the approval or admiration of less energetic colleagues, a confirmation of self-worth and protection from some of the ideas and feelings which might promote psychological pain. To become burnt out doctors need to be on fire in the first place, and it is usually these seemingly indefatigable and dedicated doctors who are most vulnerable to burn-out. The eventual costs of this extreme defence mechanism are increasing isolation from patients as people, losing touch with one's own feelings, an emotional withdrawal from family life and a cessation of non-hospital activities. Eventually interest, energy and efficiency decline and they become progressively less able to gain fulfilment from any area of professional or personal life.

Successful communication with patients demands a reasonable degree of personal awareness on the part of the doctor of the likely barriers to effective interactions. Besides negligible basic skills training few oncologists have had any guidance on means of dealing with the difficult emotions which are commonplace in oncology. Dealing with patients' emotional reactions emerged as one of the primary areas of difficulty for the 158 senior cancer clinicians attending our training courses. Providing complex information about the diagnosis, the need for further diagnostic tests and the possible therapeutic options (which could also include discussions about clinical trials) at the same time as providing reassurance or challenging the many myths and misunderstandings exacerbating distress is a daunting enough task, even for the adequately trained. Sadly, for some doctors the development of a cold, professional detachment is seen as their only means of coping with their own emotional reactions (Fallowfield, 1993).

This detachment may be protective, but also denies doctors an opportunity to engage in establishing the sorts of satisfying therapeutic relationships with their patients that make medicine so worthwhile (Fallowfield, 1992). Ramirez *et al.*, in this journal reported that 'dealing well with patients and relatives' is a great source of satisfaction for oncologists.

Perpetuation of an ethos within medicine that chronic occupational stress is a necessary initiation rite that clinicians must somehow negotiate and then maintain during their career is a sick way to work and to live. How can doctors who have been educated in a system which does not permit them to acknowledge their own feelings, who lack the knowledge and vocabulary to articulate feelings, and who have been taught to regard feelings as unimportant or a secondary issue, possibly relate appropriately with empathy and respect to the problems of their patients (Androe, 1994)?

We must do something urgently to correct these problems. There are means of teaching good communication and management skills, increasing evidence of their efficacy and doctors are keen and willing to learn these things. Will others have the vision and political will to provide resources to make these training initiatives part of post-graduate, continuing medical education?

**Acknowledgement**
The author would like to thank the Cancer Research Campaign for sponsoring the research and courses on communication skills for senior clinicians.

### References

ANDROE M. (1994). Facing death: physicians' difficulties and coping strategies in cancer care. Umea University Medical Dissertations, Uppsala, Sweden.

AUDIT COMMISSION. (1993). What seems to be the matter: communication between hospitals and patients. *NHS Report*, **12**, HMSO: London.

BRUSTER S, JARMAN B, BOSANQUET N, WESTON D, ERENS R AND DELBANCO TL. (1994). National survey of hospital patients. *Br. Med. J.*, **309**, 1542-1546.

CHRISTIE-SEELY J. (1986). Marriage and medicine: the physician as partner, parent and person. *Can. Family Physician* **32**, 360-368.

DELVAUX N, RAZAVI D AND FARVACQUES C. (1988). Cancer care - a stress for health professionals. *Soc. Sci. Med.*, **27**, 159-166.

EDITORIAL. (1994). Burnished or burnt out: the delights and dangers of working in health. *Lancet*, **344**, 1583.

FALLOWFIELD LJ. (1992). The ideal consultation. *Br. J. Hosp. Med.*, **47**, 364-367.

FALLOWFIELD LJ. (1993). Giving sad and bad news. *Lancet*, **341**, 476-478.

FALLOWFIELD LJ. (1995). Communication skills of oncologists. *Trends Exp. Clin. Med.*, **5.1**, 99-103.

FREUDENBERGER H. (1974). Staff burnout. *J. Soc. Issues*, **30**, 159.

LIPKIN M. (1987). The medical interview and related skills. In *The Office Practice of Medicine*, pp. 1287-1306. WB Saunders: Philadelphia.

McCUE JD. (1982). The effects of stress on physicians and their medical practice. *N. Engl. J. Med.*, **306**, 458-463.

REID W. (1993). *Health Service Commission Fourth Report. Session 1992 93. HC764.* HMSO: London.

SMYTH JF, MOSSMAN J, HALL R, HEPBURN S, PINKERTON R, RICHARDS M, THATCHER N AND BOX J. (1994). Conducting clinical research in the new NHS. *Br. Med. J.*, **309**, 457-461.

WHIPPEN DA AND CANELLOS GP. (1991). Burnout syndrome in the practice of oncology: results of a random survey of 1,000 oncologists. *J. Clin. Oncol.*, **9**, 1916-1920.